# Musicians’ Online Performance during Auditory and Visual Statistical Learning Tasks

**DOI:** 10.3389/fnhum.2017.00114

**Published:** 2017-03-14

**Authors:** Pragati R. Mandikal Vasuki, Mridula Sharma, Ronny K. Ibrahim, Joanne Arciuli

**Affiliations:** ^1^Department of Linguistics, Macquarie UniversitySydney, NSW, Australia; ^2^The HEARing CRC, The University of MelbourneParkville, VIC, Australia; ^3^ARC Centre of Excellence in Cognition and its Disorders, Macquarie UniversitySydney, NSW, Australia; ^4^Faculty of Health Sciences, University of SydneySydney, NSW, Australia

**Keywords:** online segmentation, auditory statistical learning, visual statistical learning, musicians, N400

## Abstract

Musicians’ brains are considered to be a functional model of neuroplasticity due to the structural and functional changes associated with long-term musical training. In this study, we examined implicit extraction of statistical regularities from a continuous stream of stimuli—statistical learning (SL). We investigated whether long-term musical training is associated with better extraction of statistical cues in an auditory SL (aSL) task and a visual SL (vSL) task—both using the embedded triplet paradigm. Online measures, characterized by event related potentials (ERPs), were recorded during a familiarization phase while participants were exposed to a continuous stream of individually presented pure tones in the aSL task or individually presented cartoon figures in the vSL task. Unbeknown to participants, the stream was composed of triplets. Musicians showed advantages when compared to non-musicians in the online measure (early N1 and N400 triplet onset effects) during the aSL task. However, there were no differences between musicians and non-musicians for the vSL task. Results from the current study show that musical training is associated with enhancements in extraction of statistical cues only in the auditory domain.

## Introduction

Long-term musical training has been associated with positive effects on the encoding of auditory information. For example, it has been reported that musicians have larger brain responses to speech (Musacchia et al., 2007), better pre-attentive discrimination of small changes in auditory stimuli (Koelsch et al., 1999) and better skills at organizing tones according to changing pitch relations (van Zuijen et al., 2004). Musicians have predictive error detection, that is to say changes in their brain responses can be observed even before they have played incorrect keystrokes (Maidhof et al., 2009; Ruiz et al., 2009). These changes in brain responses indicating predictive error detection were observed when musicians were given auditory feedback (e.g., Maidhof et al., 2009) and even when musicians could not hear what they played (i.e., independent of auditory feedback as reported by Ruiz et al., 2009). Musician’s brains are often used to examine effects of training, consequently referred to as a model of cortical plasticity (Münte et al., 2002). Hence, studying the differences in brain responses of musicians and non-musicians could assist in understanding the long-term consequences of musical training. One topic of interest is the ability to identify statistical regularities in auditory or visual input, referred to as statistical learning (SL). We investigated whether long-term musical training is associated with enhanced SL by comparing musicians and non-musicians.

SL, a form of implicit learning, is a powerful learning mechanism thought to play a key role in everyday situations such as language processing in children and adults (Kuhl, 2004; Misyak et al., 2010; Kidd and Arciuli, 2016). It was first described by Saffran et al. (1996a) who showed that infants can use statistical regularities such as transitional probabilities (TP) to segment continuous sequences of syllables. They used an embedded triplet paradigm for evaluating SL. In this paradigm, participants are first exposed to a continuous sequence of auditory or visual stimuli in which each stimulus is presented one at a time. Unbeknown to the participants, the continuous sequence is comprised of smaller sequences such as triplets. The items within a triplet have a strong statistical probability (or high transitional probability) of co-occurring. Thus, presentation of one item within a triplet strongly predicts presentation of the subsequent item. Boundaries for these “embedded” triplets occur where transitional probability between the two items is low. After being exposed to such a continuous stream for a period of time, referred to as familiarization, participants are assessed on how well they learnt the “embedded” triplets using a behavioral task. Discrimination between embedded and novel triplets is assessed using a habituation paradigm in infants and a forced-choice task in adults. Sensitivity to statistical cues (TP) helps in identification of familiar triplets due to segmentation of the continuous sequence during the familiarization phase.

In the seminal study by Saffran et al. (1996a), a stream of syllables was used for familiarization. Since then, SL has been evaluated in different modalities (auditory, visual and tactile) using a variety of stimuli. Some of the stimuli used to measure auditory SL (aSL) include speech syllables (Saffran et al., 1996b), tones (Saffran et al., 1999), morse code (Shook et al., 2013) and sung language (Schön and François, 2011). Commonly used stimuli to evaluate visual SL (vSL) include geometrical shapes (Fiser and Aslin, 2002), colored shapes (Kirkham et al., 2002) and cartoon figures (Arciuli and Simpson, 2011, 2012b). Overall, by using various types of stimuli, these studies have demonstrated the robustness of SL mechanism.

An emerging area of research is the association between implicit SL and music exposure. Although knowledge of music can be acquired explicitly, it is also acquired implicitly through attending and interacting with a large number of music samples (Rohrmeier and Rebuschat, 2012). A variety of musical structures and features can be learnt implicitly; for instance, timbre sequences (Tillmann and McAdams, 2004), chord sequences (Jonaitis and Saffran, 2009) and rhythmic patterns (Schultz et al., 2013). The familiarity of these implicitly acquired structures further governs the liking of such structures (Zajonc, 2001). Consequently, SL and wider implicit learning mechanisms may hold the key to learning musical structures and appreciation of music. Better implicit learning primes and sharpens formation of expectancies, and can help in parsing processes that underlie recognition as well as segmentation (Rohrmeier and Rebuschat, 2012).

Although it is reasonable to assume that musical training might be associated with better performance on implicit learning tasks, the experimental results do not always concur. Some behavioral studies have reported that musicians were better than non-musicians at learning statistics in a stream of morse code (Shook et al., 2013), and tone triplets (Mandikal Vasuki et al., 2016). Other studies have shown that musicians and non-musicians had similar performance for learning of unfamiliar music scales (Loui et al., 2010), and learning of a sung language (François and Schön, 2011). Most of the aforementioned studies used a behavioral measure to assess SL. Using a neurophysiological measure along with a behavioral measure can give us a deeper insight into mechanisms of SL in this population. More generally, studying these mechanisms enhances our understanding of experience-driven cortical plasticity in musicians.

SL has been studied using neurophysiological measures such as electroencephalography (EEG), magnetoencephalography (MEG) and near infrared spectroscopy. SL can be studied in two ways using these techniques. First, an online measure of SL can be obtained by recording EEG during the familiarization phase (e.g., Abla et al., 2008). Second, EEG can be recorded during the test phase so as to compare the event related potentials (ERPs) obtained in response to familiar and unfamiliar items (e.g., François and Schön, 2011). In particular, the N1-P2 and the N400 regions have been identified as neurophysiological correlates of SL (Cunillera et al., 2006, 2009; Abla et al., 2008; Abla and Okanoya, 2009).

A study by Sanders et al. (2002) investigated online measures of SL using a continuous speech stream in adult non-musicians. This stream comprised trisyllabic words (e.g., *babupu, bupada* etc.) and was used for familiarization. They showed that word onsets (i.e., initial syllables) elicited larger N1 and N400 potentials. This effect was referred to as the word onset effect. As described previously, a high within-word transitional probability makes the later items within a word more predictable/familiar. In contrast, due to low TP at word boundaries, it is difficult to predict the onset of a word (Abla et al., 2008). Thus, larger ERPs are obtained in response to unfamiliar items resulting in a word onset effect. The word onset effect is considered as evidence of successful segmentation. In a series of studies, Abla and colleagues also recorded ERPs during familiarization phase in adult non-musicians using non-linguistic stimuli. In the first study, an online measure of aSL was obtained as participants listened to three familiarization streams made by concatenation of six pure tone triplets (Abla et al., 2008). In the second study, tones were replaced by familiar, geometric shapes to assess vSL (Abla and Okanoya, 2009). Based on the performance in the subsequent test phase, participants were divided into high learners, middle learners, and low learners. The high learners showed a triplet onset effect (larger N1 and N400 for the initial stimulus of a triplet) during the first stream in the aSL task. While a triplet onset effect was observed in the later streams for the middle learners, it was absent in the low learners for all streams in the aSL task. In the vSL task, however, a triplet onset effect (larger N400) was observed in the first stream for the high learners only.

Francois and colleagues compared aSL in musicians and non-musicians using ERPs. They recorded ERPs during the test phase of an embedded triplet paradigm using sung language triplets (François and Schön, 2011). They showed that musicians exhibited a familiarity effect (smaller responses for familiar items) at around 200 ms (P2) and at later negativity (around 450 ms) for linguistic and musical stimuli. A subsequent study (François et al., 2014) recorded ERPs in musicians and non-musicians during exposure to a familiarization stream of sung language embedded triplets. To explore ongoing brain dynamics as learning takes place, the entire session was divided into four time bins and the N400 amplitude across the four time bins was compared. Both groups showed an increase in the N400 amplitude in the first time bin. The amplitude increase in both groups was attributed to building up of initial prototypes. The non-musicians showed a linear increase in the N400 amplitude across the rest of the time bins. However, in the musician group, amplitude of the N400 reached an asymptote between the second and third time bin, followed by a decrease in amplitude in the fourth time bin. Thus, an inverted U (increase-asymptote-decrease) learning curve was observed in musicians. The asymptote in the learning curve of musicians was attributed to consolidation of units into templates, while the decrease in amplitude was due to the effect of repetition of templates (familiarity effect). This finding was interpreted as faster segmentation of a sung language stream by musicians.

A study by Paraskevopoulos et al. (2012b) also compared aSL in musicians and non-musicians by recording MEG during familiarization. The familiarization task was an oddball task comprising standard musical tone triplets and oddball triplets and was designed to elicit mismatch negativity (MMN). No difference in MMN amplitude was observed between the groups. Musicians showed a larger P50 response for standard triplets when compared to the oddball triplet. Importantly, subsequent two two-alternative forced choice (AFC) behavioral trials in a separate test phase showed that neither group performed above chance level in recognizing the standard triplets. Thus, there was no musicians’ advantage observed for either MMN or the behavioral responses. The authors concluded that failure to learn the three tone patterns (i.e., the standard triplets) may be due to the complexity of stimulus patterns and very short inter-stimulus intervals.

Interestingly, some studies have demonstrated that musical training may be linked with enhancements in visual processing. For instance, musicians have been reported to have larger gray matter in areas associated with visual processing such as superior parietal cortex (Gaser and Schlaug, 2003). Further, Patston et al. (2007) used latency of N1 responses to measure interhemispheric transfer time (IHTT) in musicians and non-musicians. The IHTT represented the speed of transfer for visual information across the corpus callosum. Non-musicians showed faster IHTT from the right to the left hemisphere than from left-to-right. In contrast, the musicians showed no directional advantage indicating a more balanced visual processing in musicians than in non-musicians. A study comparing audiovisual integration in musicians and non-musicians using MEG reported larger activation in right middle frontal gyrus for the musicians in response to visually deviant stimuli (Paraskevopoulos et al., 2012a). In addition, memory for visual materials, and visual attention is also enhanced in musicians (Rodrigues et al., 2007; Jakobson et al., 2008). However, very little is known about how the learning of regularities in the visual domain (vSL) proceeds in musicians. Whilst previous studies have investigated aSL in musicians using neurophysiological paradigms, there have been few empirical investigations of both aSL and vSL in musicians.

In the present study, we compared online measures of aSL and vSL in musicians and non-musicians by recording ERPs during the familiarization phase. Specifically, we investigated how musicians and non-musicians perform on: (a) online segmentation and behavioral tasks assessing aSL; and (b) online segmentation and behavioral tasks assessing vSL. To this end, we used an embedded triplet paradigm for assessing unimodal auditory and visual SL. All participants performed both aSL and vSL tasks. Data for both aSL and vSL tasks were collected in the same session. However, for the purposes of clarity, the aSL and vSL tasks are described in different sections. To offset any potential interference effects, the order of presentation of aSL and vSL tasks was counterbalanced across participants. Regardless of the order, all participants first completed the familiarization phases for the aSL and vSL tasks. Participants then proceeded to the surprise test phase in the same order as the familiarization. That is to say, a participant who completed vSL familiarization before aSL familiarization, subsequently completed the vSL test phase before the aSL test phase.

In the present study, instead of speech syllables, we used pure tones (aSL) and cartoon figures (vSL) for familiarization. Thus, for the purposes of this study, we refer to the word onset effect as a triplet onset effect consistent with Abla and Okanoya (2009). We hypothesized that we would obtain a triplet onset effect characterized by larger ERP responses (N1 and N400) for the first stimulus compared to the third stimulus within a triplet. Note that detailed behavioral results for these same participants are published in Mandikal Vasuki et al. (2016), along with other auditory and cognitive data, but are reported again here to facilitate interpretation of ERP data. In addition, we also performed correlational analyses to examine the associations between online and behavioral measures. ERP effects may be measured through multiple independent analysis of variance (ANOVAs). However, we lose crucial information when using these methods, and the selection process of “interesting” data or “grouping electrodes” is open to user biases (Mensen and Khatami, 2013). Moreover, the multivariate nature of electrophysiological data, that is, measuring a physiological signal over a large number of electrodes, at a number of time points, increases family-wise error rates. To overcome these problems, we analyzed ERP data using a cluster-based permutation statistical analysis (Maris and Oostenveld, 2007).

## Auditory Statistical Learning (aSL)

We assessed aSL in musicians and non-musicians using pure tone triplets similar to that reported in Saffran et al. (1999). We hypothesized that musicians would show a larger triplet onset effect in the online measure of SL compared to non-musicians.

### Materials and Methods

#### Participants

Seventeen musicians (mean age of 32 years; SD 13.2) and 18 non-musicians (mean age of 28.9 years; SD 9.3) with normal hearing (defined as < = 20 dB HL pure-tone thresholds at octave frequencies from 250 Hz to 8000 Hz), normal to near normal corrected vision, and no history of neurological disorders participated in the study. An independent samples *t*-test showed that the groups did not differ significantly in age (*t*_(33)_ = 0.81 *p* = 0.42, *d* = 0.3). Musicians were classified as individuals who had learnt music before the age of nine and had more than 10 years of musical experience. All the musician participants reported that they still actively practiced music. Details about the musical and educational background of the participants have been previously described in Mandikal Vasuki et al. (2016). Participants who were categorized as non-musicians had minimal to no formal musical training, and did not report playing a musical instrument at the time or routinely participating in any musical activity (other than informal listening). Only three non-musicians reported having previous musical experience (less than 3 years, on average).

All participants lived in the greater Sydney metropolitan area, were native speakers of English and were right handed as assessed using Edinburgh Handedness inventory (Oldfield, 1971). The study was approved and conducted under the ethical oversight of the Macquarie University Human Participants Ethics Committee. Written consent was received from all participants in accordance with the Declaration of Helsinki. Subsequent to the participation, all participants were provided with a gift voucher towards their traveling expenses.

#### Stimuli and Tasks

The SL task was designed based on previously published embedded triplet tasks (Saffran et al., 1996b, 1999; Arciuli and Simpson, 2011, 2012a,b) which consisted of a familiarization phase and a separate surprise test phase.

##### Familiarization Phase

The stimuli for aSL familiarization task were created using musical tones from the same chromatic set (beginning at middle C) as previously described (Saffran et al., 1999; Abla et al., 2008). Eleven pure tones were created using MATLAB (R2013a). The tones were 550 ms in duration with 25 ms rise and fall time. Based on the previous study (Abla et al., 2008), we used 550 ms stimulus duration to obtain non-overlapping ERP responses for individual stimuli within a triplet. These tones were combined in succession to form six triplets (ADB, DFE, GG#A, FCF#, D#ED, CC#D). The six triplets were then concatenated pseudo-randomly to form three continuous streams of stimuli (e.g., ADBGG#AD#EDADBFCF#). Following previous studies (Arciuli and Simpson, 2011, 2012b), the triplets were combined with two randomization constraints: (a) consecutive repetition of a triplet was not allowed (e.g., ADBADB would not be allowed); and (b) consecutive repetition of two triplets in the same order was not allowed (e.g., ADBGG#AADBGG#A would not be allowed). Thus, the stimuli can be regarded as streams where triplets are “embedded”. Each stream was made up of 40 repetitions of a triplet. A stream was approximately 7 min in length.

There was no significant difference between the frequency of the tones at each position within tone triplets (*F*_(2, 10)_ = 1.48, *p* = 0.28). The mean frequency of tones within a triplet were as follows: ADB = 409.2 Hz; DFE = 324.2 Hz; GG#A = 415.8 Hz; FCF#= 326.9 Hz; D#ED = 311.5 Hz; and CC#D = 277.5 Hz. There was no significant difference between mean pitch intervals within- vs. across-triplets (3.1 vs. 4.9 half tones in average). The TPs within triplets ranged from 0.25 to 1 (mean 0.625) whereas the TPs across triplet boundaries were 0.04–0.3 (mean 0.11). At the end of familiarization, a participant would have been exposed to 21 min (7 min × 3 streams) of aSL stimuli.

EEG data was recorded during the familiarization phase to obtain an online measure of learning. While participants listened to the familiarization stimuli, they also performed a cover task to ensure attentiveness. The cover task was an oddball detection task. The oddball stimulus was a pure tone with a frequency of 1319 Hz. Forty presentations of the oddball stimulus occurred randomly at the end of triplets. To ensure learning was implicit, participants were neither given instructions about the nature of the embedded triplets within the familiarization stream nor told to learn or remember anything. Participants were also unaware of the upcoming test phase.

##### EEG Recording

EEG and electrooculography (EOG) signals were collected as the participants were exposed to the familiarization streams. Both horizontal (HEOG) and vertical (VEOG) signals were acquired by placing four electrodes: one at the outer canthus of each eye and one below and above the right eye. EEG was recorded using 64 electrodes set up according to the international 10–20 system (Jasper, 1958). EEG was recorded using Ag/AgCl sintered electrodes attached to EasyCap^®^ on a Neuroscan system, (Compumedics Inc. Charlotte, NC, USA). The impedance of all electrodes was maintained below 5 kΩ using a combing technique (Mahajan and McArthur, 2010). All data were sampled at 1000 Hz. Triggers were inserted to mark the onset of each stimulus within a triplet.

##### Test Phase

After the familiarization phase was completed, participants were informed about the surprise test phase. The construction of the 36 trials surprise test was based on previously published research (Saffran et al., 1999). Six novel triplets were created by combining the same previously mentioned 11 pure tones. The constituent tones in novel triplets had never occurred in that order in the familiarization phase. The task was a two-AFC task where each embedded triplet was paired with a novel triplet. The order of presentation of the embedded and novel triplets was counterbalanced. The participants were asked to indicate which of the two triplets was familiar to them through a button press response.

### Data Analysis

As reported by Mandikal Vasuki et al. (2016) a behavioral index of learning was calculated as the percentage of correctly identified embedded triplets during the test phase. Consistent with previous SL studies (Conway et al., 2010; Arciuli and Simpson, 2012a; Stevens et al., 2015), participants who scored outside the mean by ±2 SD were excluded from further analyses. One-sample *t*-tests were used to determine whether SL performance was significantly different from chance (50%) in each group. We then conducted an independent *t*-test to compare performance across the two groups (musicians and non-musicians).

ERP analysis was performed only for the participants retained after exclusion based on score deviation. The continuous EEG files were labeled according to the order of presentation—stream 1, stream 2 and stream 3. Ocular artifacts were removed using EOG artifact reduction implemented in Edit module of Neuroscan (Scan 4.5). After ocular artifact removal, EEG was further processed using Fieldtrip toolbox (Oostenveld et al., 2011) implemented in MATLAB (R2014a). The data were re-referenced to the average of the left (M1) and the right (M2) mastoids. The re-referenced signals were bandpass filtered with a frequency cut-off between 0.1 Hz and 30 Hz and a transition band roll-off of 12 dB/octave.

The filtered continuous EEG was divided into 750 ms epochs which ranged from −100 ms to 650 ms relative to the onset of the presented tone. The epochs were then baseline corrected using the mean amplitude of the signal between the −100 ms and 0 ms period. Each epoch represented the evoked response to a single stimulus in the embedded triplet. In order to remove noisy trials, a variance rejection criteria was used. Trials which had variances of more than 300 μV^2^ between −100 ms up to 650 ms were excluded from further analysis. The accepted trials were averaged to obtain the ERP waveform. To evaluate the triplet onset effect, ERP waveforms for the initial tone (T1) and final tone (T3) of embedded triplets were compared in each group.

Non-parametric randomization procedure was used to overcome the problem of multiple comparisons over a large group of electrodes (Maris, 2004; Maris and Oostenveld, 2007). The mean amplitude (μV) values for each individual stimulus in time bins of 1 ms starting from 40 ms after the onset of a trigger were taken was input. The statistical analysis produced the following outputs: a cluster of electrodes in which the difference between the conditions tested was significant in each time bin; the sum of *t* statistics in that cluster; and Monte Carlo estimates of *p-*values. The output is considered corrected for multiple comparisons as only those clusters will be identified that have higher cluster values than 95% of all clusters derived by random permutation of data. This process was implemented using the Fieldtrip toolbox and custom MATLAB scripts. Consistent with previous research (Abla et al., 2008), to compare the performance in the online segmentation (aSL) task, we measured the triplet onset effect (T1 vs. T3) for each stream in the two groups.

In order to check if the individual differences in ERP responses were associated with behavioral performance on the SL task, we performed a brain-behavior correlation for all the participants. As triplet onset effect indicates successful segmentation, we calculated the difference between ERPs evoked by the initial stimulus and final stimulus within a triplet for each stream in the SL task (T1−T3). Then, the correlation procedure implemented in BESA statistics 2.0 was applied to test the association between the difference waveform and the behavioral SL score. The inputs for this procedure were the ERP difference waveforms (0–650 ms) and the behavioral SL scores. Correction for multiple comparisons over a large group of electrodes was performed using data clustering and permutation testing (Maris and Oostenveld, 2007). This process tested for a relationship between behavioral test phase results and ERPs obtained during the familiarization phase.

### Results

#### Behavioral SL

After removal of outliers as described in previous section, a total of 34 participants were retained for aSL (17 musicians). Participants in both groups responded correctly to the oddball stimulus with over 80% accuracy. The mean performance of musicians was 78.3% (SD 7.8) and non-musicians was 67.2% (SD 11.5) on the behavioral aSL task. Individual subject data can be visualized in Mandikal Vasuki et al. (2016; see Figure 3). Both groups performed significantly above chance on the aSL task (one sample *t*-test; musicians *t*_(16)_ = 14.9, *p* < 0.001, *d* = 3.62; non-musicians *t*_(16)_ = 6.1, *p* < 0.01, *d* = 1.49). The behavioral SL scores were normally distributed as assessed by Shapiro-Wilk’s test (*p* > 0.05). There was homogeneity of variances, as assessed by Levene’s test for equality of variances (*p* > 0.05). An independent samples *t*-test showed that musicians outperformed non-musicians in the aSL task (*t*_(32)_ = 3.29, *p* < 0.01, *d* = 1.13). In addition, an item analysis showed that responding was consistent across all the six triplets in both groups.

#### Event Related Potentials

**Figure 1:**
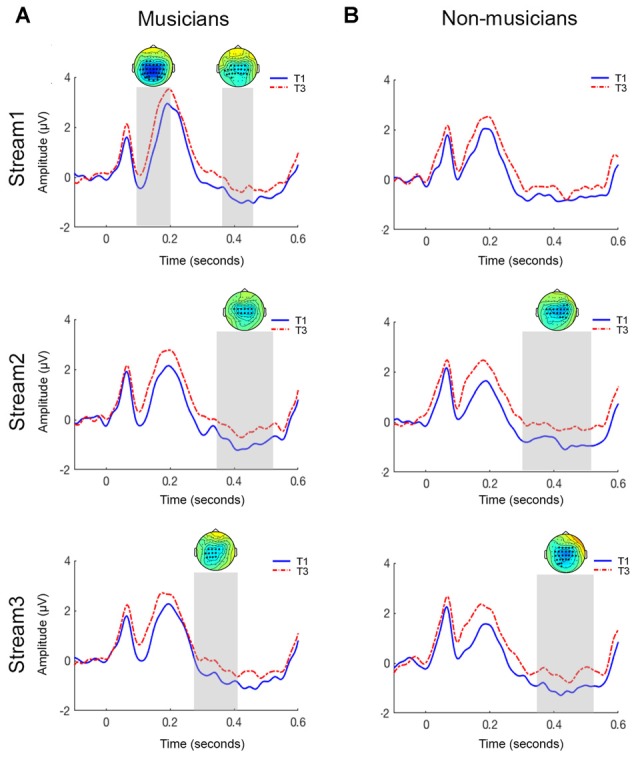
**Grand-averaged ERP waveforms for central electrodes sites for musicians (A)** and non-musicians **(B)** in the auditory statistical learning (aSL) online task. The topoplots show significant clusters between initial tone (T1) and final tone (T3) of a triplet. Top panel = stream 1; middle panel = stream 2; bottom panel = stream 3.

ERPs were used to obtain an online measure of segmentation capability in musicians and non-musicians. Figure 1 shows the grand-averaged ERP waveforms and topographies elicited in response to initial (T1) and final (T3) tones of a triplet across the three streams for musicians and non-musicians. In both groups, we observed an N1 component peaking at approximately 100 ms, and a P2 component peaking at approximately 200 ms followed by an N400 component between 300 ms and 500 ms.

##### Musicians

The amplitude of the N1 component was significantly larger for T1 than T3 in the first stream (*p* < 0.01). The significant clusters were distributed centrally. This difference was not observed in streams 2 and 3 (all contrasts *p* > 0.05).

An N400 triplet onset effect, where amplitude of the N400 was larger for T1 than T3, was observed in all three streams (stream 1: *p* < 0.05; stream 2: *p* < 0.05; stream 3: *p* < 0.05). As seen in Figure 1, cluster permutation statistics showed significant clusters (represented by asterisks) between 380 ms and 470 ms with a centro-parietal distribution.

##### Non-musicians

In contrast to the musicians, there was no significant difference in the amplitude of the N1 component for T1 and T3 across all three streams (all contrasts; *p* > 0.05; Figure 1).

Testing for an N400 triplet onset effect in the latency range from 350 ms to 500 ms post-stimulus, the cluster-based permutation test revealed no significant difference between T1 and T3 in stream 1 (*p* > 0.05). Interestingly, a significant difference was observed for this latency range in streams 2 and 3 (stream 2: *p* < 0.05; stream 3: *p* < 0.05). This effect was most pronounced over the centro-parietal electrodes.

#### Brain-Behavior Correlation

**Figure 2:**
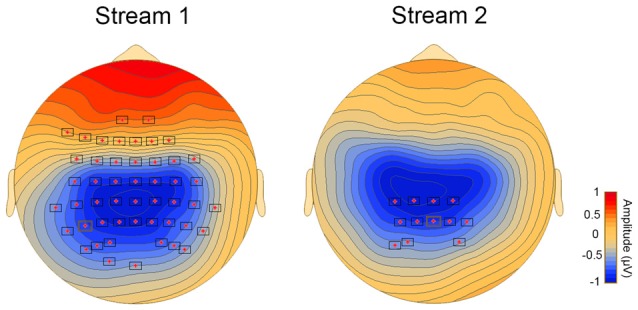
**Topoplots showing cluster of electrodes where significant correlations were obtained between behavioral and online measures of aSL**.

Correlational analysis revealed a significant association between the difference waveform, that is ERPs for T1−T3 and the behavioral SL performance in streams 1 and 2 (stream 1 and 2: *p* < 0.05). The significant clusters with a central distribution were found between 150 ms and 300 ms (Figure 2).

### Discussion

We hypothesized that musicians would show a larger triplet onset effect in the online measure of aSL compared to non-musicians. Data analysis revealed that, when compared with non-musicians, musicians were better able to segment a continuous tone stream during the ERP task. Better performance on the ERP task was characterized in musicians by presence of both N1 and N400 triplet onset effects during the initial part of familiarization (stream 1). An interesting finding is the appearance of the N400 triplet onset effect only in the later parts of familiarization (streams 2 and 3) for the non-musician group. Consistent with previous literature (Sanders et al., 2002, 2009), the N400 triplet onset effect was distributed in the centro-parietal regions.

In the current study, musical pure tones were used to construct embedded triplets. In order to recognize that the continuous streams were made up of these embedded triplets, participants relied on TPs. Participants in both groups were able to use these cues and learn the embedded triplets. However, musicians outperformed non-musicians. Better performance of musicians in the behavioral aSL task may indicate that long-term musical training primes musicians to form associations between successive stimuli and thus helps them in making better use of the TPs. This interpretation is supported by a recent longitudinal study which reported that children learning music for 2 years can significantly improve their aSL abilities when compared to a control group (François et al., 2013). However, the present findings must be interpreted as evidence of an association only—our design does not allow us to determine causality.

We used ERPs to obtain an online measure of segmentation in this population. When listening to continuous sequences of sounds, elements in the initial part of a sequence elicit a larger negativity. Reduction of both N1 and N400 components for predictable stimuli has been reported to be a “marker” or “index” for online segmentation of continuous sound sequences (Sanders et al., 2002, 2009). The presence of the N1 and N400 triplet onset effects could arise from one of the two possibilities: (i) differences in processing of initial and final tones of a triplet; or (ii) the process of segmentation itself. Findings from Sanders et al. (2009) study show that the N1 and N400 triplet onset effects cannot be solely attributed to acoustic differences between stimuli. Moreover, in our stimuli there was no significant difference between the frequencies of tones at each position within the triplets. The second explanation is supported by research that shows that the triplet onset effect reflects sensitivity to statistical regularities and not acoustic differences between the stimuli (Astheimer and Sanders, 2011). Moreoever, the centro-parietal distribution of the ERP effects is consistent with an interpretation of the N400 as an index of lexical search (Laszlo and Federmeier, 2011).

Interestingly, only the musician group showed an N1 triplet onset effect during the early part of the familiarization sequence (stream 1). Previous studies have shown that the N1 triplet onset effect may be seen in the participants who were classified as “expert” or “high” learners based on performance in the behavioral task (Sanders et al., 2002, 2009). These findings were attributed to use of additional resources such as selective attention during learning. Additionally, Abla et al. (2008) also reported the presence of an N1 triplet onset effect for their high learner group (i.e., those who performed above mean + 0.5 SD on the behavioral task) only in stream 1. Taken together, these findings suggest that musicians, when compared with non-musicians, perform as “high learners” or “expert listeners” and possibly use additional strategies such as selective attention for successful recognition of statistical regularities.

Additionally, only the musicians exhibited the N400 triplet onset effect in the first stream. This finding indicates that musicians were able to utilize the statistical structure of the stream and could segment it faster than non-musicians. There are several explanations for these findings. At the beginning of the familiarization, all items of the triplet are novel. As each item is heard, an increase in exposure presumably helps in formation of the triplet (three-tone) template by computation of TPs. Working memory resources are helpful during the consolidation of these templates (Cunillera et al., 2009; Lopez-Barroso et al., 2011). Recent research also suggests that SL is supported by working memory process which may include attentional refreshing or working memory update (Palmer and Mattys, 2016). Due to consolidation of the three-tone templates, tones within a triplet are easy to predict while the first tone of the triplet continues to be unpredictable resulting in a strong triplet onset effect. It is plausible that individuals who demonstrate faster update of working memory resources may be at the advantage in SL tasks. Not surprisingly, research has shown that musicians demonstrate faster update of working memory (George and Coch, 2011) and increased neural activity during working memory tasks (Pallesen et al., 2010). In addition, previous studies have shown that musicians are also better at grouping and processing complex auditory patterns (van Zuijen et al., 2004; Boh et al., 2011). Thus, it is plausible that musicians may be relying more on these abilities than non-musicians to achieve segmentation.

The N400 component is also regarded as an indicator of successful segmentation of the continuous sequence (Cunillera et al., 2009). The appearance of the N400 triplet onset effect in the later streams (streams 2 and 3) in non-musicians may indicate that non-musicians require larger periods of familiarization to successfully segment sequences. This finding is consistent with the ERP effects seen in the middle learner group during the previous study by Abla et al. (2008). Overall, the appearance of N400 triplet onset effect in early streams in musicians and in the later streams in the non-musicians further supports the notion that musicians may be faster at segmentation tasks (i.e., detection of statistical regularities).

## Visual Statistical Learning (vSL)

The mechanisms underlying online segmentation of visual stimuli were explored by replacing auditory stimuli with visual stimuli for the same participants. Following reports of musicians’ enhanced performance on visual tasks (Jakobson et al., 2008; Anaya et al., 2017), we hypothesized that musicians would outperform non-musicians in the online vSL task.

### Materials and Methods

#### Participants

All participants completed both aSL and vSL tasks.

#### Stimuli and Tasks

Similar to the aSL paradigm, an embedded triplet task was used to assess vSL. The SL task consisted of a familiarization and a surprise test phase.

##### Familiarization Phase

**Figure 3:**
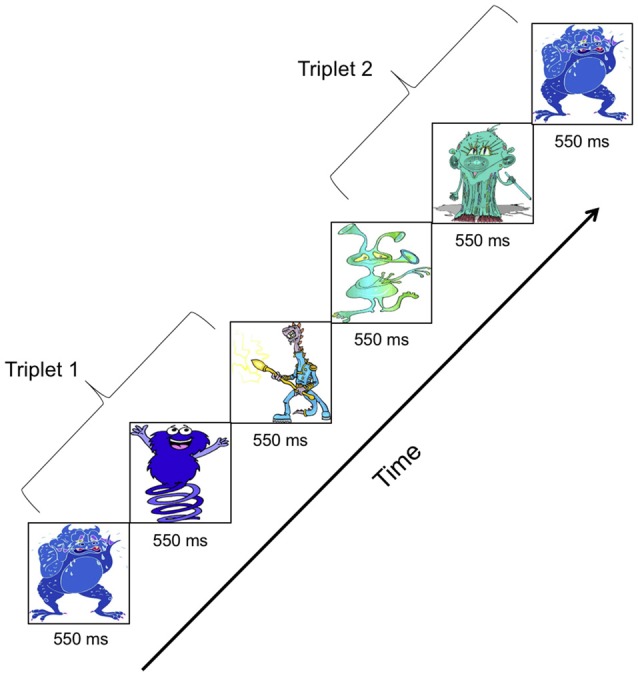
**Familiarization stimuli for visual statistical learning (vSL) showing presentation of two cartoon figure “embedded” triplets**.

The vSL task was designed identically to the aSL task. Eleven cartoon-like figures best described as aliens used by Arciuli and colleagues (see appendix in Arciuli and Simpson, 2011) replaced the 11 pure tones used to construct aSL stimuli. The cartoon figures were rescaled to have equal height and width. These cartoon figures were combined to form six triplets. The six triplets were concatenated pseudo-randomly to form the three familiarization streams. Each stream contained 240 triplets (40 repetitions × 6 triplets) and was 7 min long. Figure 3 illustrates the presentation of two triplets during a vSL familiarization stream. The vSL stimuli were delivered using Presentation software^1^ on a CRT monitor placed 1 m away from the participant. Each cartoon figure was presented for 550 ms against a black background. The vSL familiarization streams had identical statistical structure as the aSL streams. An oddball detection cover task was used where participants were asked to press a button every time they saw a particular cartoon figure. A total of 40 presentations of the oddball stimulus occurred randomly at the end of triplets within each stream. EEG was recorded while participants watched the familiarization streams.

##### EEG Recording

The procedure and instrumentation for recording EEG was identical to that described under the aSL task. Triggers were inserted to mark the onset of each cartoon figure within a triplet.

##### Test Phase

A 2-AFC test phase with 36 trials was constructed in which embedded cartoon figure triplets were paired with novel cartoon figure triplets that had never occurred during familiarization. The novel triplets were made up of the same 11 cartoon figures but had never occurred in that order during the familiarization. Participants had to indicate through button press response the triplet that they thought had been presented during familiarization.

#### Data Analysis

For the behavioral vSL task, statistical analysis including removal of outliers was performed using the same procedures as described under the aSL task.

The continuous EEG files were labeled according to the order of presentation—stream 1, stream 2 and stream 3. The ERP analysis followed the same steps as described under the aSL task. To evaluate the triplet onset effect, ERPs elicited to first picture (P1) and third picture (P3) of a triplet were compared in each stream for musicians and non-musicians. Analysis of vSL surprise test phase results and the brain-behavior correlation was performed similar to the aSL task.

### Results

#### Behavioral SL

After removal of outliers, a total of 32 participants were retained for analysis (16 musicians). All participants detected the oddball stimulus with an accuracy of above 80%. The mean performance of musicians was 54.3% (SD 6.5) and non-musicians was 55.5% (SD 7.6) on the behavioral vSL task. See individual participants’ data in Mandikal Vasuki et al. (2016; Figure 3). Performance on the vSL task was significantly above chance for both groups (one sample *t*-test; musicians *t*_(15)_ = 2.7, *p* < 0.05, *d* = 0.67; non-musicians *t*_(15)_ = 2.9, *p* < 0.05, *d* = 0.73). The behavioral SL scores were normally distributed as assessed by Shapiro-Wilk’s test (*p* > 0.05). There was homogeneity of variances, as assessed by Levene’s test for equality of variances (*p* > 0.05). An independent samples *t*-test showed that both groups performed similarly on the vSL task (*t*_(30)_ = −0.49, *p* > 0.05, *d* = 0.17).

#### Event Related Potentials

**Figure 4:**
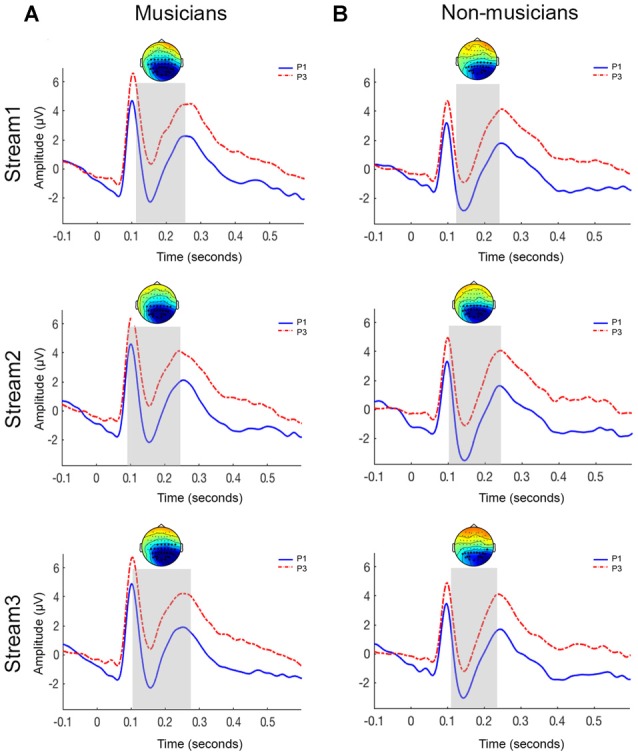
**Grand-averaged ERP waveforms for parieto-occipital electrodes sites for musicians (A)** and non-musicians **(B)**. The topoplots show significant clusters between initial picture (P1) and final picture (P3) of a triplet. Top panel = stream 1; middle panel = stream 2; bottom panel = stream 3.

Figure 4 shows the grand-averaged ERP waveforms and topographies elicited in response to initial (P1) and third (P3) pictures within a triplet across the three streams for musicians and non-musicians. In both groups, a P1 component peaking at approximately 100 ms, an N1 component peaking at approximately 150 ms and a P2 component peaking at approximately 250 ms was seen. The N400 component was not seen in ERPs for either P1 or P3 stimuli. After selecting the a-priori time of interest in our data (N1-P2 region), the cluster-based permutation tests were applied to evaluate the triplet onset effect.

##### Musicians

The N1-P2 response to P1 was significantly larger than the response to the P3 across all the three streams (stream 1: *p* < 0.005; stream 2: *p* < 0.05; stream 3: *p* < 0.001). Figure 4 shows the significant clusters (represented by asterisks) over the parieto-occipital and occipital electrodes.

##### Non-musicians

A significant difference was observed between the P1 and P3 stimuli across all three streams (stream 1: *p* < 0.001; stream 2: *p* < 0.005; stream 3: *p* < 0.005). Similar to the musician group, this effect was observed over the parieto-occipital and occipital electrodes (Figure 4).

#### Brain-Behavior Correlation

**Figure 5:**
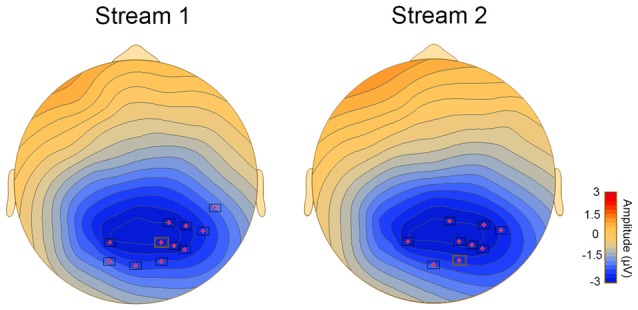
**Topoplots showing cluster of electrodes where significant correlations were obtained between behavioral and ERP measures of vSL**.

Behavioral performance on the vSL task was significantly correlated with the difference waveform obtained by subtracting the ERPs for P1 minus the ERPs for P3 stimulus in streams 1 and 2 only (*p* < 0.05). Significant clusters with a parieto-occipital distribution were found between 150 ms and 300 ms (Figure 5).

### Discussion

We investigated whether musical expertise was associated with better performance during vSL. We hypothesized that musicians would outperform non-musicians. Our hypothesis was rejected as musicians and non-musicians had similar performance during vSL. The N400 response was not seen in the ERPs to visual stimuli in either group. However, both groups showed a significant reduction in the N1-P2 amplitude as a function of stimulus position, that is, larger N1-P2 responses for the P1 compared to the P3 within a triplet.

We evaluated vSL using an embedded triplet paradigm where the individual stimuli were colored cartoon figures. Learning of these visual “embedded” triplets was significantly above chance in both groups. It is interesting to note that the mean behavioral performance for both groups were lower than those reported in a study using a similar paradigm (Abla and Okanoya, 2009). One explanation for this could be that we used unknown cartoon figures (abstract shapes i.e., aliens) in our vSL instead of familiar, nameable, geometric shapes that were used for vSL in the Abla study. Thus, for the shapes used in our study, participants could not rely on a verbal encoding strategy during learning. It has been speculated that if the stimuli can be verbalized participants may use cues other than statistical cues for learning (for further discussion see, Conway and Christiansen, 2005 experiments 1B and 1C). There is some evidence in the literature that musicians exhibit advantages in processing of visual stimuli. For example, better design learning (Jakobson et al., 2008) and sequential learning of visuo-spatial patterns (Anaya et al., 2017) has been reported in musicians. However, we did not observe a musicianship advantage in our cohort for learning of visuo-temporal sequences.

While Abla and Okanoya (2009) reported the presence of an N400 triplet onset effect between the ERP responses for first and third shape of the shape-words used in their study, we did not observe an N400 triplet onset effect in our dataset. There are two possible explanations for the difference in ERP morphologies between the two studies. First, the appearance of specific ERP components may depend on difficulty level of the stimuli used. Comparison of mean performance of subjects in the behavioral task in the two studies (current study all participants: 54.95%; Abla and Okanoya, 2009: 72.2%) suggests that the vSL task used in the current study may be more difficult. ERP components have been shown to be related to task difficulty and task threshold (e.g., see Caryl and Harper, 1996). An alternative explanation for different ERP morphologies in the two studies could be related to the use of black and white images (e.g., Abla and Okanoya, 2009) vs. colored images (current study). The current study used complex and colored images (cartoon figures) for eliciting visual ERPs. ERP responses obtained for rapid presentation of colored images typically present with a positivity (around 100 ms), followed by a negativity (around 175–200 ms) and another broad positivity (centered around 250 ms) in the posterior electrode sites (e.g., Schupp et al., 2004). A similar pattern was observed in the ERPs in the current study (Figure 4). Overall, the significant brain-behavioral correlations over the parieto-occipital region suggest that, regardless of the group, amplitude changes in the N1-P2 region index successful segmentation of sequentially presented visual stimuli (at least for the stimuli used here).

## General Discussion

In this study, we explored online measures of SL using unimodal auditory and visual embedded triplet paradigms. As reported previously by Mandikal Vasuki et al. (2016), while musicians outperformed non-musicians on the behavioral aSL task, no group differences were seen in the behavioral vSL task. A similar pattern of results was seen in the online measures of SL. Musicians or “high” learners showed both N1 and N400 triplet onset effects in the early part of familiarization streams (stream 1) in the aSL paradigm. An N1 triplet onset effect (but not an N400 triplet onset effect) was seen in both groups for the vSL paradigm. Taken together, these findings suggest differential processing of auditory stimuli in our aSL task in individuals with musical training.

An important aspect of the current study is that the two groups of participants learnt the statistics of the stream above chance in the aSL and the vSL tasks. This is in contrast to the previous aSL studies in which awareness of the statistics of the stream was not demonstrated in one or both groups (in particular the non-musician group; François and Schön, 2011; Paraskevopoulos et al., 2012b; Shook et al., 2013; François et al., 2014). One possible reason for this could be due to complexity of the aSL task in these studies. For example, François and Schön (2011) and François et al. (2014) used sung language for familiarization. However, they used purely linguistic (i.e., no pitch contour cues) or purely musical (no linguistic cues) stimuli for the subsequent behavioral 2AFC task. A second reason could be that participants’ knowledge of statistical information may not be stable enough to be demonstrated through behavioral measures (e.g., see Koelsch et al., 2016). Nevertheless, the results of the present study demonstrate that musicians’ advantage in behavioral aSL task is not due to fact that the aSL stimuli were too difficult to be learnt by the non-musicians.

Increased exposure to sounds and auditory training through repeated practice may sharpen musicians’ processing of sounds. This could facilitate better and faster segmentation of auditory stimuli where probabilistic cues are key to segmentation. This could be reflected as larger differences in the ERP components for initial tones than final tones within a triplet, as well as higher scores on the aSL triplet recognition task. However, an alternative explanation could be that the ERP effects observed in the current study are due to modulation of the level of attention (Daltrozzo and Conway, 2014). Indeed, in a previous study, musicians displayed enhanced top-down modulatory attentional effects in ERPs elicited to auditory stimuli (Tervaniemi et al., 2009). Moreover, another study showed that musicians recruit more neuronal networks that sustain attention and cognitive control (Pallesen et al., 2010). The enhancements in attention and cognitive control may be used to better integrate information during segmentation tasks compared to non-musicians. These explanations may not necessarily be mutually exclusive but may operate in a complementary manner thereby facilitating segmentation of auditory stimuli in musicians.

Although music is a multimodal learning experience, enhanced SL was only seen in one modality (auditory not visual). Studies in artificial grammar learning also report limited transfer of learning across modalities (Tunney and Altmann, 1999). However, caution must be observed when comparing results across modalities because it is very difficult to control the perceptual saliency of the aSL and vSL tasks. Our vSL task was based on probabilistic sequences in the visuo-temporal domain. Previous studies have shown that the visual sense is more adept at processing spatial cues (Conway and Christiansen, 2005, 2009). A recent study showed that musicians may be more adept at extracting statistical information in visuo-spatial stimuli (Anaya et al., 2017). Perhaps tasks involving online processing (ERPs) of probabilistic visuo-spatial sequences could further explore the neurophysiological mechanisms of vSL in individuals with musical expertise.

In summary, our findings show that musical training is associated with enhanced sensitivity to statistical regularities in auditory stimuli. These enhancements were also observed in the online measure of SL using ERPs. By measuring both neurophysiological and behavioral indices of SL in auditory and visual modalities, our findings add to the growing literature on musical expertise and performance on SL tasks.

## Author Contributions

PRMV, MS, JA designed the study. PRMV collected the data. PRMV, RKI analyzed the data. PRMV wrote the article. All authors contributed in revising the article critically for intellectual content.

## Conflict of Interest Statement

The authors declare that the research was conducted in the absence of any commercial or financial relationships that could be construed as a potential conflict of interest.

## Footnotes

^1^www.neurobs.com
